# Comparison of fludarabine/melphalan (FluMel) with fludarabine/melphalan/BCNU or thiotepa (FBM/FTM) in patients with AML in first complete remission undergoing allogeneic hematopoietic stem cell transplantation – a registry study on behalf of the EBMT Acute Leukemia Working Party

**DOI:** 10.1038/s41409-023-02150-w

**Published:** 2023-12-02

**Authors:** Jesús Duque-Afonso, Jürgen Finke, Maud Ngoya, Jacques-Emmanuel Galimard, Charles Craddock, Kavita Raj, Adrian Bloor, Emma Nicholson, Matthias Eder, Orchard Kim, Thomas Valerius, John A. Snowden, Eleni Tholouli, Charles Crawley, Matthew Collin, Keith M. O. Wilson, Alain Gadisseur, Rachel Protheroe, Eva Maria Wagner-Drouet, Bipin N. Savani, Alexandros Spyridonidis, Fabio Ciceri, Arnon Nagler, Mohamad Mohty

**Affiliations:** 1 Department of Hematology/Oncology, Faculty of Medicine, University of Freiburg Medical Center, Freiburg, Germany; 2 EBMT Statistical Unit, INSERM UMRs 938, Hôpital Saint Antoine, Paris, France; 3 Birmingham Centre for Cellular Therapy and Transplantation, Queen Elizabeth Hospital Birmingham, Edgbaston, Birmingham, UK; 4 Department of Haematology, University College London Hospital, London, UK; 5 The Christie NHS Foundation Trust, Stem Cell Transplantation Unit, University of Manchester, Manchester, UK; 6 Department of Haematology, Royal Marsden Hospital, London, UK; 7 Department of Haematology, Hannover Medical School, Hemostasis, Oncology and Stem Cell Transplantation, Hannover, Germany; 8 Department of Haematology, Southampton General Hospital, Haematology, Oncology & Paediatrics, Southampton, UK; 9 Department of Medicine II, University Medical Center Schleswig-Holstein, Campus Kiel, Section of Stem Cell Transplantation and Immunotherapy, Kiel, Germany; 10 Department of Hematology, Sheffield Teaching Hospitals NHS Trust, Royal Hallamshire Hospital, Sheffield, UK; 11 Clinical Haematology Department, Manchester Royal Infirmary, Manchester, UK; 12 Department of Haematology, Addenbrookes Hospital, Cambridge, UK; 13 Adult HSCT unit, Northern Centre for Bone Marrow Transplantation, Freeman Hospital, Newcastle Tyne, UK; 14 Department of Haematology, University Hospital of Wales, Cardiff, UK; 15 Department of Hematology, Antwerp University Hospital (UZA), Antwerp Edegem, Belgium; 16 University Hospitals Bristol and Weston NHS Foundation Trust, Bristol, UK; 17 Department of Hematology, University Medical Center Mainz, Oncology and Pneumology, Mainz, Germany; 18 Division of Hematology and Oncology, Vanderbilt University Medical Center, Nashville, TN USA; 19 University Hospital of Patras, Patras, Greece; 20 University Vita-Salute, IRCCS San Raffaele Scientific Institute, Milan, Italy; 21 Hematology Division, Chaim Sheba Medical Center, Tel Hashomer, Israel; 22 Sorbonne University, Saint-Antoine Hospital, AP-HP, INSERM UMRs 938, Paris, France

**Keywords:** Translational research, Stem-cell research

## Abstract

Conditioning protocols for patients undergoing allogeneic hematopoietic cell transplantation (allo-HCT) are being developed continuously to improve their anti-leukemic efficacy and reduce their toxicity. In this study, we compared the conditioning protocol of fludarabine with melphalan 140 mg/m^2^ (FluMel) with conditioning protocols based on this same backbone but with an additional alkylating agent i.e., either fludarabine/BCNU (also known as carmustine)/melphalan (FBM), or fludarabine/thiotepa/melphalan (FTM) 110 mg/m^2^. We included 1272 adult patients (FluMel, *n* = 1002; FBM/FTM, *n* = 270) with acute myeloid leukemia (AML) with intermediate/poor cytogenetic risk in first complete remission (CR) from the registry of the EBMT Acute Leukemia Working Party. Despite patients in the FBM/FTM group were older (64.1 years vs. 59.8 years, *p* < 0.001) and had a worse Karnofsky performance score (KPS < 90, 33% vs. 24%, *p* = 0.003), they showed a better overall survival (OS) (2 y OS: 68.3% vs. 58.1%, *p* = 0.02) and less non-relapse mortality (NRM) (2 y NRM: 15.8% vs. 22.2%, *p* = 0.009) compared to patients treated with FluMel. No significant differences were observed in relapse incidence (RI) (2 y RI: 24.9% vs. 23.7%, *p* = 0.62). In conclusion, the addition of a second alkylating agent (BCNU/carmustine or thiotepa) to FluMel as FBM/FTM conditioning, improves OS in AML patients in first CR with intermediate/poor risk cytogenetics after allo-HCT.

## Introduction

Conditioning protocols for patients undergoing allogeneic hematopoietic cell transplantation (allo-HCT) are developing continuously and are assessed for efficacy and toxicity. Conditioning regimens have been intensified for increased killing of leukemia cells, but this has increased the risk of short- and long-term toxicities and high-intensity regimens cannot be tolerated by older patients [1]. The conditioning intensity has been shown to be a continuum in previous studies and a novel transplant conditioning intensity (TCI) score has been established [2]. In this study we compared from the ‘intermediate’ TCI score the conditioning protocol of fludarabine with the single alkylating agent melphalan (FluMel) with conditioning protocols based on this same backbone but with an additional alkylating agent i.e., either fludarabine/BCNU (also known as carmustine)/melphalan (FBM), or fludarabine/thiotepa/melphalan (FTM).

Conditioning with FluMel is a standard conditioning protocol in many centers and has been showed to have moderate toxicity and good anti-leukemic activity [3, 4]. Patients treated with FluMel have a similar relapse incidence than patients treated with classical myeloablative ablative conditioning (MAC) protocols Bu4Cy and FluBu4 and they show a lower relapse incidence than reduced intensity conditioning (RIC) protocol FluBu2 [4]. Lower relapse incidence and higher non-relapse mortality (NRM) was shown for patients receiving FluMel compared to patients treated with the lower-intensity conditioning protocol fludarabine/busulfan (FluBu2), although similar rates of leukemia-free survival (LFS) and overall survival (OS) were observed in patients with AML [5]. However, improved LFS and OS were observed in patients with MDS treated with FluMel compared to FluBu2 [6]. Using the registry of the Acute Leukemia Working Party (ALWP) from the European Society for Blood and Marrow Transplantation (EBMT), it was previously shown that FluMel had conferred a lower relapse rate, but higher NRM compared to patients conditioned with fludarabine/treosulfan (FluTreo) in patients with AML in complete remission (CR). However, patients had similar outcomes regarding OS and LFS [7].

To improve the anti-leukemic effect of conditioning without increasing toxicity, protocols combining two alkylating agents had been developed in the last few decades. For example, the FBM conditioning protocol was developed by adding BCNU to FluMel conditioning and by reducing the melphalan dose [8]. The conditioning protocol FBM has shown low toxicity and remarkable anti-leukemic activity not only in AML patients with active disease, but also in the second transplantation setting and in patients with multiple comorbidities [9–11]. Similarly, the FTM conditioning protocol was established by adding thiotepa to the FluMel backbone. Haplo-identical allo-HCT after conditioning with the FTM protocol has been well tolerated and effective for patients with AML/myelodysplastic syndromes (MDS) with low tumor burden [12, 13] and as a conditioning protocol for patients with multiple myeloma [14]. Hence, previous studies have shown that both conditioning protocols based on two alkylating agents (FBM or FTM) have adequate anti-leukemic activity and were suitable for older patients or those with co-morbidities including impaired lung function. Hence, both protocols were comparable regarding adjusted OS [15, 16].

In this registry-based study, we hypothesized that patients treated with conditioning based on the fludarabine and melphalan backbone with the addition of a second alkylating agent as BCNU (FBM) or thiotepa (FTM) and undergoing allo-HCT for AML with intermediate/poor cytogenetics in first CR would have better outcomes than patients receiving FluMel as conditioning.

## Patient and methods

### Study design

In this retrospective multicenter analysis, data were provided by the ALWP of the EBMT, a scientific society representing >600 transplant centers, mainly in Europe who report annually all consecutive allo-HCTs after patient authorization via informed consent, and approval of the study by the general assembly of the ALWP of the EBMT. We focused on (1) adult (aged >18 years) patients who received conditioning with FluMel (fludarabine, median 150 mg/m^2^; melphalan 140 mg/m^2^) or with FBM (fludarabine, median 150 mg/m^2^; BCNU 300–400 mg/m^2^ and melphalan 110 mg/m^2^) or with FTM (fludarabine, median 150 mg/m^2^; thiotepa 5–10 mg/kg and melphalan 110 mg/m^2^), (2) first allo-HCT from a matched sibling donor (MSD) or unrelated donor for patients with AML including secondary AML (secAML) in first CR, (3) AML with intermediate/poor cytogenetics risk, (4) transplantation date between January 1st, 2009 and December 31st, 2020, (5) with an unmanipulated peripheral blood graft (no in vitro T-cell depletion (TCD) and no bone marrow grafts). Patients undergoing haploidentical allo-HCT were excluded. Three hundred eighteen (25%) of the patients received an unrelated donor for which the HLA was incomplete or low resolution making impossible to calculate the high resolution mismatches on A, B, C, DRB1 and DQB1.

In previous single-center-based studies, it was shown that FBM and FTM protocols are comparable after adjusting for variables influencing mortality in multivariate analysis [16]. This was confirmed using EBMT registry-based data in a preliminary analysis of the present study. For this reason and knowing that BCNU and thiotepa are both alkylating agents, we decided to include FBM- and FTM-treated patients in the same group.

### Statistical analysis

Outcome variables were defined following internal consensus guidelines [17]. Patient-, disease- and treatment-related characteristics were compared using the chi-square test for categorical data or the Mann–Whitney test for continuous data. Baseline characteristics were summarized using median, interquartile range (IQR), and range, for continuous data, and frequency and percentage for categorical data. OS was defined as the time from allo-HCT until death from any cause. LFS was defined as the time from allo-HCT to death from any cause, or relapse/progression, whichever occurred first. Relapse was defined as detection of disease via cytological and histological assessment after allo-HCT; death without prior relapse was considered as a competing risk for relapse and was denoted as NRM. For cumulative incidence of acute graft-versus-host disease (aGvHD) and chronic GvHD (cGvHD), death without aGvHD/cGvHD and relapse were considered as competing events. GvHD-free, relapse-free survival (GRFS) was defined as being alive with neither grade III–IV aGVHD nor severe cGVHD, relapse, or death from any cause post-HCT. Patients with no event were censored at the date of last follow-up. To allow for the difference in follow-up period between the 2 conditioning regimen groups, outcome was censored at 2 years post transplantation for all comparisons.

Univariate analyses were performed using Gray’s test for cumulative incidence functions and the log-rank test for OS, GRFS, and LFS. The Cox proportional-hazards model was used for multivariable regression analysis and included variables with unbalanced distribution between the two groups or factors known to predict outcomes. To allow for center differences, a random effect or frailty was introduced for each center into the models. Results were expressed as the hazard ratio (HR) with the 95% confidence interval (95% CI).

All tests were two sided. The Type I error was fixed at 0.05 for factors associated with time-to-event outcomes. Statistical analyses were performed with R 3.6.1 (R Development Core Team, Vienna, Austria) software packages.

## Results

### Patient and transplant characteristics

The patient and transplant characteristics of the 1272 AML patients are shown in Table 1. Prior to allo-HCT, 1002 (79%) patients received a conditioning with FluMel, and 270 (21%) patients received FBM/FTM. Before censoring at 2 years, the median follow-up was 2.6 years (95% CI, 2.2–2.9) in the FluMel and 2.0 years (95%CI, 1.6–2.2 years) in the FBM/FTM group, respectively. Patients in the FBM/FTM group were older compared to patients in the FluMel group (median age of 64.1 years vs. 59.8 years, p < 0.001). Compared with FluMel patients, those conditioned with FBM/FTM showed a significantly worse performance, with KPS < 90 (33% vs. 24% in FluMel, *p* = 0.003). Additional transplant characteristics such as secondary AML (21% vs. 14% in FluMel, *p* = 0.003), donor type (81% vs. 66% unrelated in FluMel, *p* < 0.0001) varied significantly between the groups. GvHD prophylaxis based on in vivo T-cell depletion (TCD) was similar between both groups (91% vs 92% in FluMel, *p* = 0.39) but the type of in vivo TCD differed significantly (82% ATG in FBM/FTM and 74% alemtuzumab in FluMel, *p* < 0.0001) (Table 1).

**Table 1:** Patient and transplant characteristics.

Variable	Entire cohort	FluMel	FBM/FTM	*p*-value
N (%)	1272 (100)	1002 (79%)	270 (21%)	
Year of allo-HCT				
‐ median (range)	2017 (2009–2020)	2017 (2009–2020)	2017 (2009–2020)	0.71
‐ IQR	2015–2019	2015–2019	2016–2018	
Median Follow-up	2.3	2.6	2.0	
(years) [95%CI]	[2.1–2.7]	[2.2–2.9]	[1.6–2.2]	
Conditioning (%)				
‐ FluMel	1002 (79%)	1002 (100%)		
‐ FBM	220 (17%)		220 (81%)	
‐ FTM	50 (4%)		50 (19%)	
Melphalan dose				
‐ median (range)	140 (110–144.4)	140 (135–144.4)	110 (110–115)	<0.001
‐ [IQR]	[138.4–140]	[140–140]	[110–110]	
BCNU dose (for FBM)				
‐ 300 mg/m^2^	218 (99%)		218 (99%)	
‐ 400 mg/m^2^	2 (1%)		2 (1%)	
Thiotepa dose (for FTM)				
‐ 10 mg/kg	35 (70%)		35 (70%)	
‐ 5 mg/kg	15 (30%)		15 (30%)	
Patient age (years)				<0.001
median (min-max)	61.2 (20.5–76.4)	59.8 (21.1–75.2)	64.1 (20.5–76.4)	
[IQR]	[54.4–65.5]	[52.5–64.8]	[60.4–66.9]	
Age group				<0.0001
‐ age <61 years	629 (49%)	555 (55%)	74 (27%)	
‐ age ≥61 years	643 (51%)	447 (45%)	196 (73%)	
KPS score				0.0034
‐ <90	302 (25%)	218 (24%)	84 (33%)	
‐ ≥90	881 (75%)	707 (76%)	174 (67%)	
‐ missing	89	77	12	
Patient sex				0.69
‐ female	567 (45%)	444 (44%)	123 (46%)	
‐ male	703 (55%)	557 (56%)	146 (54%)	
‐ missing	2	1	1	
Donor sex				0.72
‐ female	404 (32%)	321 (32%)	83 (31%)	
‐ male	859 (68%)	675 (68%)	184 (69%)	
‐ missing	9	6	3	
Female to male combination				0.1
‐ No	1064 (84%)	830 (83%)	234 (87%)	
‐ Yes	202 (16%)	168 (17%)	34 (13%)	
‐ missing	6	4	2	
AML diagnosis				0.0025
‐ de novo	1078 (85%)	865 (86%)	213 (79%)	
‐ secondary AML	194 (15%)	137 (14%)	57 (21%)	
Cytogenetics				0.96
‐ intermediate	958 (75%)	755 (75%)	203 (75%)	
‐ adverse	314 (25%)	247 (25%)	67 (25%)	
Patient CMV				0.95
‐ neg	477 (38%)	375 (38%)	102 (38%)	
‐ pos	786 (62%)	619 (62%)	167 (62%)	
‐ missing	9	8	1	
Donor CMV				0.54
‐ neg	632 (50%)	503 (51%)	129 (49%)	
‐ pos	623 (50%)	487 (49%)	136 (51%)	
‐ missing	17	12	5	
Donor type				<0.001
‐ MSD	397 (31%)	345 (34%)	52 (19%)	
‐ UD	875 (69%)	657 (66%)	218 (81%)	
Donor type				<0.001
‐ MSD	397 (31%)	345 (34%)	52 (19%)	
‐ UD 10/10	465 (37%)	379 (38%)	86 (32%)	
‐ UD 9/10	92 (7%)	73 (7%)	19 (7%)	
‐ UD missing/ incomplete HLA	318 (25%)	205 (20%)	113 (42%)	
GvHD prophylaxis				n.d.
‐ CsA	719 (57%)	704 (70%)	15 (6%)	
‐ CsA + MMF	297 (23%)	109 (11%)	188 (70%)	
‐ CsA + MTX	127 (10%)	106 (11%)	21 (8%)	
‐ Other	126 (10%)	82 (8%)	44 (16%)	
‐ missing	3	1	2	
In vivo TCD				
‐ no in vivo TCD	102 (8%)	77 (8%)	25 (9%)	0.39*
‐ in vivo TCD	1168 (92%)	924 (92%)	244 (91%)	
‐ *ATG*	401 (32%)	179 (18%)	222 (82%)	<0.001**
‐ *alemtuzumab*	767 (60%)	745 (74%)	22 (9%)	
‐ missing	2	1	1	

*FluMel* fludarabine/melphalan, *FBM* fludarabine/BCNU/melphalan, *FTM* fludarabine/thiotepa/melphalan, *Allo-HCT* allogeneic hematopoietic cell transplantation, *MSD* matched sibling donor, *UD* unrelated donor, *HLA* human leukocyte antigen, *AML* acute myeloid leukemia, *KPS* Karnofsky performance status, *CMV* cytomegalovirus, *neg* negative, *pos* positive, *CsA* cyclosporine A, *MTX* methotrexate, *MMF* mycophenolate mofetil, *TCD* T-cell depletion, *ATG* anti-thymocyte globulin, *GvHD* graft-versus-host disease, *NA* not assessed, *IQR* interquartile range, *CI* confidence interval.

*Statistical difference between with and without in vivo TCD. **Statistical difference between ATG vs alemtuzumab.

### Analysis of outcomes in patients conditioned with FluMel compared to FBM/FTM

**Fig. 1:**
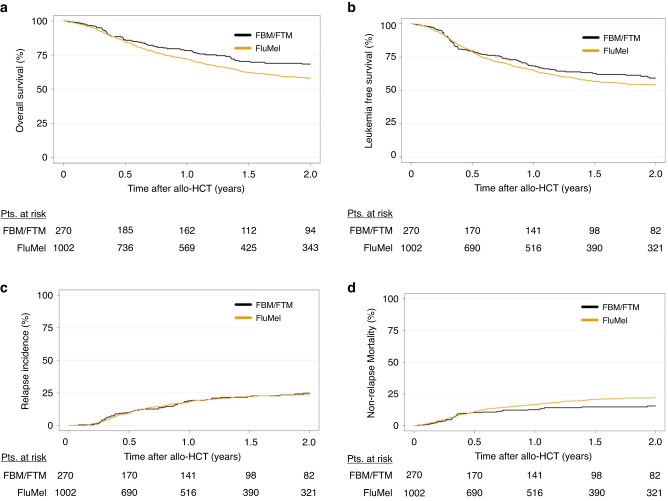
Impact of conditioning by FluMel and FBM/FTM on outcome. Kaplan–Meier curves represent (**a**) overall survival and (**b**) leukemia-free survival by conditioning protocol. Cumulative incidences of (**c**) non-relapse mortality and (**d**) relapse by conditioning protocol. FluMel, fludarabine/melphalan; FBM, fludarabine/BCNU/melphalan; FTM, fludarabine/thiotepa/melphalan; Pts, patients; allo-HCT, allogeneic hematopoietic cell transplantation.

Results are shown in Tables 2–3 and Fig. 1. According to the univariate analysis (Table 2 and Fig. 1), patients conditioned with FBM/FTM had compared to patients treated with FluMel a better OS (2 y 68.3% vs. 58.1%, HR 0.7, 95%CI 0.52–0.94, *p* = 0.02), improved LFS (2 y: 59.4% vs. 54.1%, HR 0.78, 95%CI 0.61–0.99, *p* = 0.04) and decreased NRM (2 y: 15.8% vs. 22.2%, HR 0.56, 95%CI 0.36–0.86, *p* = 0.009) (Table 3). Several clinical parameters adversely influenced OS such as age (per 10 years) at allo-HCT (HR 1.19, 95%CI 1.05–1.35, *p* = 0.006), unrelated donor (HR 1.61, 95%CI 1.27–2.04, *p* < 0.001), poor cytogenetics (HR 1.71, 95%CI 1.39–2.1, *p* < 0.001) and KPS (HR for KPS < 90 1.43, 95%CI 1.13–1.79, *p* = 0.002) (Table 3).

**Table 2:** Univariate analysis for outcome variables according to conditioning protocol.

Outcomes at 2 years	Entire cohort	FluMel	FBM/FTM
	Estimate (95%CI)	Estimate (95%CI)	Estimate (95%CI)
OS	60.1% (57–63.1)	58.1% (54.5–61.5)	68.3% (61.3–74.3)
LFS	55.2% (52.0–58.2)	54.1% (50.6–57.5)	59.4% (52.2–65.8)
RI	23.9% (21.4–26.6)	23.7% (20.8–26.6)	24.9% (19.1–31.1)
NRM	20.9% (18.5–23.4)	22.2% (19.4–25.1)	15.8% (11.2–21.0)
aGvHD II–IV (100d)	25.5% (23.1–28.0)	22.3% (19.7–25.0)	36.9% (31.0–42.7)
aGvHD III–IV (100d)	9.0% (7.5–10.7)	7.5% (5.9–9.3)	14.4% (10.5–19.0)
cGvHD	32.8% (29.9–35.7)	32.3% (29.1–35.6)	34.5% (28.2–41.0)
cGvHD Ext.	10.3% (8.5–12.3)	9.8% (7.8–12.0)	12.4% (8.2–17.5)
GRFS	45.9% (42.8–48.9)	45.9% (42.5–49.4)	45.7% (38.8–52.4)

*OS* overall survival, *LFS* leukemia-free survival, *RI* relapse incidence, *NRM* non-relapse mortality, *aGvHD* acute graft-versus-host disease, *cGvHD* chronic graft-versus-host disease, *Ext* extensive, *GRFS* GvHD-/relapse-free survival, *FluMel* fludarabine/melphalan, *FBM* fludarabine/BCNU/melphalan, *FTM* fludarabine/thiotepa/melphalan, *CI* confidence interval, *d* day, *y* year.

*Outcomes were censored at 2 years except aGvHD, which was censored at 100 days.

**Table 3:** Multivariate analysis of outcome variables.

		OS		LFS		RI		NRM		GRFS	
		HR (95% CI)	*p* value	HR (95% CI)	*p* value	HR (95% CI)	*p* value	HR (95% CI)	*p* value	HR (95%CI)	*p* value
Conditioning	FluMel	1		1		1		1		1	
	FBM/FTM	0.7 (0.52–0.94)	0.02	0.78 (0.61–0.99)	0.04	1.09 (0.78–1.52)	0.62	0.56 (0.36–0.86)	0.009	0.99 (0.8–1.22)	0.94
Year at allo-HCT (by 3 years)		0.89 (0.81– 0.99)	0.04	0.93 (0.85–1.03)	0.15	0.88 (0.78–1)	0.06	0.98 (0.85–1.14)	0.76	0.96 (0.88–1.05)	0.4
Age at allo-HCT (per 10 years)		1.19 (1.05–1.35)	0.006	1.09 (0.98–1.23)	0.12	0.89 (0.78–1.03)	0.12	1.46 (1.21–1.76)	<0.001	1.11 (0.99–1.23)	0.06
Female to Male donor	No	1		1		1		1		1	
	Yes	1.27 (0.98–1.65)	0.06	1.27 (1–1.61)	0.053	1.23 (0.88–1.71)	0.22	1.36 (0.95–1.94)	0.09	1.25 (1–1.56)	0.046
Type of donor	MSD	1		1		1		1		1	
	UD	1.61 (1.27–2.04)	<0.001	1.52 (1.22–1.9)	<0.001	1.16 (0.87–1.55)	0.32	2.17 (1.52–3.09)	<0.001	1.25 (1.03–1.53)	0.03
CMV donor	Neg.	1		1		1		1		1	
	Pos.	1.04 (0.84–1.3)	0.69	1.08 (0.88–1.33)	0.46	1.12 (0.85–1.48)	0.43	1.06 (0.78–1.43)	0.73	1.06 (0.88–1.28)	0.51
CMV patient	Neg.	1		1		1		1		1	
	Pos.	1.12 (0.89–1.41)	0.34	1.04 (0.84–1.29)	0.71	0.97 (0.73–1.3)	0.84	1.1 (0.8–1.51)	0.57	1.02 (0.84–1.24)	0.82
In vivo T cell depletion	No	1		1		1		1		1	
	Yes	0.87 (0.59–1.27)	0.47	0.92 (0.65–1.31)	0.66	1.17 (0.72–1.9)	0.53	0.75 (0.44–1.28)	0.29	0.68 (0.51–0.92)	0.01
Cytogenetics	Intermediate	1		1		1		1		1	
	Poor	1.71 (1.39–2.1)	<0.001	1.79 (1.48–2.16)	<0.001	2.41 (1.87–3.1)	<0.001	1.28 (0.95–1.73)	0.1	1.57 (1.32–1.88)	<0.001
Secondary AML	No	1		1		1		1		1	
	Yes	1.21 (0.93–1.56)	0.15	1.22 (0.96–1.56)	0.1	0.88 (0.61–1.29)	0.52	1.58 (1.14–2.18)	0.005	1.2 (0.96–1.49)	0.1
KPS	≥90	1		1		1		1		1	
	<90	1.43 (1.13–1.79)	0.002	1.41 (1.16–1.72)	<0.001	1.27 (0.96–1.68)	0.09	1.67 (1.22–2.29)	<0.001	1.38 (1.15–1.65)	<0.001

Center effect or “frailty” was taken into account. Year of allo-HCT was included as integer and not as continuous variable. The HR of year of allo-HCT was calculated corresponding to an increase of 3 years. Patients were censored at 2 years of follow up.

*FluMel* fludarabine/melphalan, *FBM* fludarabine/BCNU/melphalan, *FTM* fludarabine/thiotepa/melphalan, *LFS* leukemia-free survival, *OS* overall survival, *RI* relapse incidence, *NRM* non-relapse mortality, *GRFS* GvHD-/relapse-free survival, *HR* hazard ratio, *CI* confidence interval, *AML* acute myeloid leukemia, *CR* complete remission, *MSD* matched sibling donor, *UD* unrelated donor, *KPS* Karnofsky performance status, *Pat*. patient, *Don*. donor, *CMV* cytomegalovirus, *CR* complete remission, *neg*. negative, *pos*. positive, *NA* not assessed.

Conditioning with FluMel or FBM/FTM did not significantly influence either the cumulative incidence of relapse (2 y: 24.9% vs. 23.7%,HR 1.09, 95%CI 0.78–1.52, *p* = 0.62) (Table 3), aGvHD II–IV (100d: 36.9% vs. 22.3%, HR 1.42, 95%CI 0.94–2.15, *p* = 0.09), aGvHD III–IV (100d: 14.4% vs. 7.5%, HR 1.59, 95%CI 0.89–2.84, *p* = 0.11) or cGvHD (2 y: 34.5% vs. 32.3%, HR 0.82, 95%CI 0.61–1.11, *p* = 0.19) (Supplementary Table 1) in multivariable analysis. Cause of death was not significantly different in patients undergoing allo-HCT conditioned with FluMel compared to FBM/FTM (Supplementary Table 2).

## Discussion

Due to the increasing age (and associated comorbidities) of patients undergoing allo-HCT, conditioning protocols are in continuous development to increase their anti-leukemic effect whilst not increasing toxicity [18]. Besides the success of unrelated donor and haploidentical allo-HCT as well as the rise in cellular therapy, the use of RIC in older patients is one of the most notable developments in allo-HCT field during the last decade [19, 20]. The most frequently used conditioning protocols are based on a purine analogue with immunosuppressive effects combined with an alkylating agent with myelosuppressive properties and stem cell toxicity. Some examples based on this combination are fludarabine/busulfan with a high TCI score [21–23], and fludarabine/treosulfan [24] and fludarabine/melphalan [25] with an intermediate TCI score.

One approach to improve the anti-leukemic effect (without adversely affecting toxicity) is to add a second alkylating agent and decrease the dosage of the first alkylating substance. With this approach, an additive/synergistic effect can be expected and several modern conditioning protocols have been developed such as thiotepa (8–15 mg/kg b.w.)/fludarabine (3–4 × 40 mg/m^2^)/treosulfan (3 × 12–14 mg/m^2^) [TFTreo] [26–28] as well as thiotepa (1–2 × 5 mg/kg b.w.)/busulfan (1–3 × 3.2 mg/kg b.w.)/fludarabine (3 × 50 mg/m^2^) [TBF] [29–31] as RIC and MAC protocols. Recent data show that patients treated with two alkylating agent chemotherapies (TBF) have less relapse incidence but the same NRM as patients treated with one alkylating agent chemotherapy (BuFlu or BF) [32]. In a registry-based EBMT study, TBF-MAC and TBF-RIC showed better anti-leukemic activity but higher NRM compared to BF-MAC and BF-RIC, resulting in similar OS [33]. However, intensification of RIC conditioning protocols as FLAMSA-Bu has not resulted in reduction of cumulative incidence of relapse or improved OS and disease-free survival compared to other fludarabine-based conditioning (FluMel + alemtuzumab, FluBu2 + alemtuzumab, FluBu2 + ATG) in randomized controlled clinical trials [34].

Using FluMel as a backbone, two conditioning protocols based on a two-alkylating agent approach have been developed by adding BCNU (carmustine) (FBM) [9] or by adding thiotepa (FTM) [12]. Both protocols have been shown to have good anti-leukemic activity and lower toxicity. However, they have not been compared to FluMel conditioning in clinical trials or in large registry-based studies to demonstrate improved outcome and similar toxicity.

In this study, we analyzed patient characteristics, outcomes, and cause of death in a large registry-based study on behalf of the ALWP of the EBMT including 1272 patients with AML with intermediated/poor cytogenetics in first CR. Patients conditioned with FBM/FTM showed better OS and LFS, and lower NRM despite the patients being older, having a worse KPS and receiving a graft from unrelated donors. All these features were associated with worse OS on multivariable analysis. We only included patients receiving standard melphalan dose in FluMel of 140 mg/m^2^ and in FBM/FTM of 110 mg/m^2^. So, we did not just compare the addition of a second alkylating agent to FluMel in our study but also the reduction of melphalan dose, which probably contributed to reducing the toxicity of the FBM/FTM conditioning protocols, while the addition of the second alkylating agent maintains the anti-leukemic activity, as seen by similar relapse incidence in both conditioning groups.

Older patients and with reduced performance score received two-alkylating based protocols FBM/FTM in our cohort than the one-alkylating based protocol FluMel. We interpret these data as center- or region-dependent. Of note, there were regional differences in the use of conditioning protocols, which might reflect differences in patient populations and transplantation procedures. Patients conditioned with FluMel were treated in the United Kingdom (74.9%), Belgium (9.4%), Germany (4.7%) and Czech Republic (3.8%). However, patients conditioned with FBM/FTM were treated predominantly in Germany (99.3%).

This study has several limitations. We analyzed data from patients conditioned with FluMel and FBM/FTM, included in the EBMT registry. Due to the retrospective nature of the study, patient cohorts were not balanced with respect to patient characteristics and transplant conditions, which could have influenced on outcomes despite the adjustment in multivariable models. Although a randomized controlled trial would be the gold standard to elucidate which conditioning protocol is the most suitable for which patient population, such studies are very challenging to perform due to the relatively small number of patients. In another hand, the registry nature of this analysis reflects the real practice of the centers. Nevertheless, although patients transplanted with FBM/FTM were older and had a worse KPS, which frequently negatively influences outcomes, they showed prolonged OS. Differences in GvHD prophylaxis were observed in both cohorts, which might have influenced GvHD incidence and, hence, patient outcomes. There were no differences in the use of in vivo TCD between groups (92% in FluMel and 91% in FBM/FTM, *p* = 0.39). However, patients conditioned with FluMel more often received an in vivo TCD based on alemtuzumab (74%) compared with patients conditioned with FBM/FTM, who received more frequently ATG (82%, *p* < 0.001). No statistically significant differences were observed in the incidence of aGvHD II–IV and III–IV and cGvHD. However, we cannot exclude that differences on the substances employed (alemtuzumab or ATG) for in vivo TCD may have had an impact on OS influencing other post allo-HCT complications. It has been described that in vivo TCD as GvHD prophylaxis with alemtuzumab in combination FluMel-based conditioning is associated with higher likelihood of infectious complications as well as mixed chimerism, for which a proportion of these patients received DLI [35, 36]. In addition, data about gene mutations or MRD status, which might have impacted patient outcome, were not available in the EBMT registry for the statistical analysis. Other variables influenced by regional differences and not included in our EBMT registry as patient selection or supportive care modalities may have impacted the outcome of patients after conditioning with FluMel or FBM/FTM. Therefore, our data should be interpreted with caution in the absence of randomized controlled clinical trials.

In conclusion, despite older age, worse KPS and receiving more often grafts from unrelated donors, the addition of a second alkylating agent (BCNU/carmustine or thiotepa) to FluMel as FBM/FTM conditioning improves OS in patients with AML with intermediate/poor risk cytogenetics in first CR.

### Supplementary information

Supplementary tables

## Data Availability

The datasets generated during and/or analyzed during the current study are available upon reasonable request from the corresponding authors.
