# Supplementary material for: Comparison of fludarabine/melphalan (FluMel) with fludarabine/melphalan/BCNU or thiotepa (FBM/FTM) in patients with AML in first complete remission undergoing allogeneic hematopoietic stem cell transplantation – a registry study on behalf of the EBMT Acute Leukemia Working Party
Source: Bone Marrow Transplant. 2023 Dec 2;59(2):247–54. doi: 10.1038/s41409-023-02150-w (PMC10849951; doi:10.1038/s41409-023-02150-w)
Supplement: Supplementary file 1 — Supplementary tables [file 41409_2023_2150_MOESM1_ESM.docx]

**Suppl. Table 1.**

|  |  | **aGvHD II-IV** | | **aGvHD III-IV** | | **cGvHD** | |
| --- | --- | --- | --- | --- | --- | --- | --- |
|  |  | **HR**  **(95% CI)** | **p value** | **HR**  **(95% CI)** | **p value** | **HR**  **(95% CI)** | **p value** |
| **Conditioning** | **FluMel** | 1 |  | 1 |  | 1 |  |
|  | **FBM/FTM** | 1.42  (0.94-2.15) | 0.09 | 1.59  (0.89-2.84) | 0.11 | 0.82  (0.61-1.11) | 0.19 |
| **Year at allo-HCT**  **(by 3 years)** | | 0.98  (0.86-1.12) | 0.76 | 1.12  (0.9-1.39) | 0.31 | 1.24  (1.1-1.41) | <0.001 |
| **Age at allo-HCT**  **(per 10 years)** | | 1.06  (0.91-1.24) | 0.45 | 1.13  (0.86-1.49) | 0.37 | 1.07  (0.93-1.22) | 0.34 |
| **Female to Male donor** | **No** | 1 |  | 1 |  | 1 |  |
|  | **Yes** | 1.17  (0.85-1.6) | 0.34 | 1.11  (0.65-1.9) | 0.69 | 0.89  (0.66-1.21) | 0.47 |
| **Type of donor** | **MSD** | 1 |  | 1 |  | 1 |  |
|  | **UD** | 1.72  (1.27-2.34) | <0.001 | 1.23  (0.74-2.03) | 0.42 | 0.98  (0.75-1.28) | 0.87 |
| **CMV donor** | **Neg.** | 1 |  | 1 |  | 1 |  |
|  | **Pos.** | 1.01  (0.77-1.31) | 0.97 | 1.01  (0.65-1.56) | 0.98 | 0.99  (0.77-1.27) | 0.92 |
| **CMV patient** | **Neg.** | 1 |  | 1 |  | 1 |  |
|  | **Pos.** | 0.9  (0.68-1.19) | 0.47 | 0.86  (0.54-1.36) | 0.51 | 1.09  (0.84-1.41) | 0.52 |
| ***In vivo* T cell depletion** | **No** | 1 |  | 1 |  | 1 |  |
|  | **Yes** | 0.4  (0.27-0.6) | <0.001 | 0.41  (0.22-0.75) | 0.004 | 1.38  (0.86-2.2) | 0.18 |
| **Cytogenetics** | **Intermediate** | 1 |  | 1 |  | 1 |  |
|  | **Poor** | 0.86  (0.65-1.14) | 0.3 | 0.93  (0.58-1.47) | 0.74 | 1.29  (1.03-1.63) | 0.03 |
| **Secondary AML** | **No** | 1 |  | 1 |  | 1 |  |
|  | **Yes** | 1.2  (0.89-1.62) | 0.23 | 1.57  (0.99-2.49) | 0.056 | 1.21  (0.91-1.6) | 0.19 |
| **KPS** | **<90** | 1 |  | 1 |  | 1 |  |
|  | **≥90** | 0.83  (0.61-1.13) | 0.25 | 1.34  (0.84-2.13) | 0.22 | 1.3  (1.02-1.65) | 0.04 |

**Suppl. Table 1. Multivariate analysis of outcome variables.** GvHD, graft-versus-host disease; FluMel, fludarabine/melphalan; FBM, fludarabine/BCNU/melphalan; FTM, fludarabine/thiotepa/melphalan; aGvHD, acute graft-versus-host disease; cGvHD, chronic graft-versus-host disease; HR, hazard ratio; CI, confidence interval; AML, acute myeloid leukemia; MSD, matched sibling donor; UD, unrelated donor; KPS, Karnofsky performance score; Don., donor; CMV, cytomegalovirus; neg., negative; pos., positive; NA, not assessed. Centre effect or “frailty” was taken into account. Year of allo-HCT was included as integer and not as continuous variable. The HR of years of allo-HCT was calculated corresponding to an increase of 3 years. Patients were censored at 2 years of follow up.

**Suppl. Table 2**

|  | **Entire cohort** | **FluMel** | **FBM/FTM** | **p-value** |
| --- | --- | --- | --- | --- |
| **N** | **1272** | **1002** | **270** |  |
| Total deaths (% of patients) | 476 (37) | 392 (41) | 84 (31) |  |
| Allo-HCT related (%) | 198 (44) | 161 (44) | 37 (46) | 0.97 |
| Relapse or progression of original disease (%) | 189 (42) | 156 (42) | 33 (41) |  |
| Secondary malignancy (%) | 16 (4) | 13 (3) | 3 (4) |  |
| Other (%) | 44 (10) | 37 (10) | 7 (9) |  |
| Missing (%) | 29 | 25 | 4 |  |

**Suppl. Table 2. Cause of death.** N, total patient number; allo-HCT, allogeneic hematopoietic cell transplantation; FluMel, fludarabine/melphalan; FBM, fludarabine/BCNU/melphalan; FTM, fludarabine/thiotepa/melphalan.
